# Role of impulse oscillometry in chronic obstructive pulmonary disease and asthma‐chronic obstructive pulmonary disease overlap

**DOI:** 10.1002/clt2.70057

**Published:** 2025-04-22

**Authors:** Yuning Huang, Xue Zhang, Jinwen Wang, Wuping Bao, Chengjian Lv, Yingying Zhang, Xue Tian, Yan Zhou, Min Zhang

**Affiliations:** ^1^ Department of Respiratory and Critical Care Medicine Shanghai General Hospital Shanghai Jiao Tong University School of Medicine Shanghai China

**Keywords:** acute exacerbation, asthma‐COPD overlap impulse oscillometry, small airway dysfunction, spirometry

## Abstract

**Background:**

Small airway dysfunction (SAD) is critical in chronic obstructive pulmonary disease (COPD) and asthma‐COPD overlap (ACO), impacting disease severity, acute exacerbation (AE) risk, and prognosis. Traditional spirometry may miss SAD due to its reliance on forced vital capacity.

**Objective:**

This study investigates the role of impulse oscillometry system (IOS) for early detection, disease monitoring, and AE prediction.

**Methods:**

Pathological specimens from 64 patients with normal lung function were divided into small airway pathological abnormalities (PAs, *n* = 38) and normal pathology (PN, *n* = 26). Logistic regression and receiver operating characteristic (ROC) curve analysis evaluated IOS's predictive value for SAD. Additionally, 37 healthy volunteers, 125 COPD patients, and 128 ACO patients underwent spirometry, IOS, FeNO, CT scans, and blood tests. Correlations between IOS and spirometry indices were evaluated. One‐year follow‐up of 140 patients assessed IOS's predictive capability for AE.

**Results:**

ROC analysis indicated that R5 − R20 combined with FEF_75_%pred best predicted PAs (areas under the ROC curves [AUC] = 0.80). R5 − R20, with a cut‐off of 0.09 kPa/[L/s], demonstrated 85.6% sensitivity and 72.9% specificity in distinguishing COPD from healthy individuals, and 89.1% sensitivity with 72.9% specificity for ACO. In COPD, R5 − R20 correlated strongly with spirometry indices (*r* = 0.60), while Fres correlated well in ACO (*r* = 0.48) for FEV_1_%pred ≥ 50%, with slightly weaker correlations for FEV_1_%pred < 50%. For predicting AE, a model combining R5 − R20, FEV_1_%Pred and body mass index had an AUC of 0.860 in COPD, while a model with Fres, FEV_1_%pred and fraction of exhaled nitric oxide achieved an AUC of 0.874 in ACO.

**Conclusions:**

IOS is valuable for early detection, monitoring, and AE prediction in COPD and ACO, enhancing diagnostic precision.

**Clinical Trial Registration:**

No. ChiCTR2400089625, www.chictr.org.cn.

## 1 INTRODUCTION

Chronic obstructive pulmonary disease (COPD) and asthma‐COPD overlap (ACO) are both characterized by persistent airflow limitation, often presenting with lower forced expiratory volume in 1 s (FEV_1_) and reduced lung function over time. In COPD, the airflow limitation is progressive and associated with an abnormal inflammatory response to noxious particles, primarily affecting the small airways and leading to destruction of alveolar structures.^1^ ACO is a commonly encountered clinical condition in chronic airway diseases, characterized by features typically associated with both asthma and COPD, such as asthma with fixed airflow obstruction.^2^, ^3^, ^4^ Despite ongoing debate over whether ACO represents a distinct clinical entity or part of a disease spectrum, these conditions, although distinct in their pathophysiology, share a common feature: small airway dysfunction (SAD).

Small airways, <2 mm in diameter, are essential for gas exchange but vulnerable to inflammation, mucus secretion, and alveolar damage, contributing to chronic airway disease progression.^5^, ^6^ Detecting airway obstruction and early pathological changes in small airways is the key to improving outcomes. Traditional diagnostic methods such as spirometry have limitations in identifying SAD as they rely heavily on forced vital capacity (FVC). In patients with reduced FVC, forced expiratory flow measurements may be significantly affected, leading to an underestimation of small airway involvement.^7^ Additionally, COPD patients may have SAD even before spirometry detects abnormalities.^8^ While a decline in FEV_1_ can signal increased exacerbation risk in COPD and asthma, it is difficult to monitor in older or non‐compliant patients. This underscores the need for more sensitive and convenient tools like the impulse oscillometry system (IOS).^9^ IOS, increasingly utilized in both clinical practice and medical research, does not require the effort of forced expiration, which can affect small airway closure.^10^ IOS differentiates increases in airway resistance between central and peripheral obstruction using sound signals at 5 and 20 Hz.^11^ It measures total (R5), proximal (R20), and peripheral airway resistance (R5 − R20), while X5 reflects lung compliance, and Fres indicates where reactance balances inertia and capacitance.^12^ These features make it a valuable complement to spirometry, especially for patients who cannot perform it.^13^

Despite these capabilities, the role of IOS in the early diagnosis of SAD remains to be fully elucidated, particularly regarding the optimal cutoff values for detecting early pathological changes. Furthermore, while IOS shows potential in predicting acute exacerbations (AE) in chronic airway diseases, it raises the question of whether there are differences in predictive indicators for AE across various airflow‐limiting conditions.^14^ This uncertainty limits its application in clinical settings, where precise and reliable markers are essential for early intervention and effective disease management.^15^ Therefore, further research is needed to validate these aspects and enhance the clinical utility of IOS in managing COPD and ACO.

This study aims to explore the utility of IOS in detecting early structural abnormalities in patients with obstructive airway diseases. We first undertook a pathological validation to ascertain the small airway changes. This initial step ensures the reliability of IOS in reflecting true structural alterations. Following this, a cohort study was conducted to observe the clinical characteristics across different patient groups, providing comprehensive insights into the utility of IOS in routine clinical practice. By validating the efficacy of IOS indices, particularly R5 − R20, in a cohort of 125 COPD patients and 128 ACO patients, we sought to establish IOS as a complementary tool.

## 2 METHODS

### 2.1 Part I

#### 2.1.1 Study design

This retrospective study was approved by the ethics committee of Shanghai General Hospital (No. 2020KY014). Sixty‐four consecutive patients who underwent thoracotomy for pulmonary nodules in the Department of Thoracic Surgery from November 2018 to October 2019 had preserved FEV_1_ and FEV_1_/FVC ratios. Inclusion criteria were: aged 18–70 years, pulmonary nodules ≤3 cm, eligibility for lobectomy, FEV_1_/FVC ≥ 70%, FEV_1_ > 80% predicted, and negative bronchodilation test (FEV_1_ increase <200 mL and improvement rate <12%). Exclusion criteria included: (1) respiratory tract infection in the past 8 weeks; (2) lung infection history with atelectasis on CT; (3) abnormal CT results aside from nodules; And other details are as mentioned before.^16^

#### 2.1.2 Assessment of histopathological features of small airway

Inflammatory cell counts, alveolar spaces, and alveolar septa were measured from histological sections. To prevent bias, slides were coded and analyzed blindly by two pathologists. Cell counts were measured as the mean number of cells per square millimeter (i.e., 1 mm^2^) measured at three different sections. The air space size, as denoted by the mean linear intercept, was measured using a line grid in the QCapture Pro 6.0 software developed by QImaging Inc., headquartered in Surrey, British Columbia, Canada. The final result was the mean of all measurements obtained for each biopsy specimen. Final results averaged all measurements, and samples were classified into two groups based on total scores assigned to inflammatory cells and alveolar intervals.^17^, ^18^, ^19^

### 2.2 Part II

#### 2.2.1 Study design

In this retrospective study, a total of 290 patients—comprising 37 healthy volunteers, 125 patients with COPD and 128 patients with ACO—were consecutively enrolled between January 2021 and October 2023. All participants underwent spirometry, IOS, high‐resolution computed tomography (HRCT) scans, fraction of exhaled nitric oxide (FeNO) measurement, and blood cell analysis. This retrospective study was approved by the ethics committee of Shanghai General Hospital (No. ChiCTR2400089625).

#### 2.2.2 Participants

All enrolled patients were stable with normal cardiac function and no significant lung lesions on HRCT. The diagnosis of COPD was based on the Global Initiative for Chronic Obstructive Lung Disease (GOLD) guidelines, with inclusion criteria of age ≥40, ≥10 pack‐year smoking history, persistent respiratory symptoms, and post‐bronchodilator FEV_1_/FVC <70%. ACO diagnosis followed the guidelines outlined by the Global Initiative for Asthma involving the presence of current episodic respiratory symptoms and confirmed by evidence of variable expiratory airflow limitation (positive bronchodilator response in bronchodilator test). Healthy controls had normal spirometry and no chronic respiratory symptoms. Exclusion criteria included elevated inflammation, abnormal liver or kidney function, recent infections, other lung diseases, systemic conditions, recent bronchodilator use, pregnancy, or fever.

#### 2.2.3 Assessment of AE

Patients were followed up for 1 year via telephone or clinic visits to monitor AE. We defined asthma AE as the requirement for systemic corticosteroids for more than 3 days and/or hospitalization, consistent with the European Respiratory Society (ERS)/American Thoracic Society (ATS) guidelines.^20^ The AE of COPD was defined as an acute episode of worsening respiratory symptoms that required additional treatment. Mild exacerbations were managed with short‐acting bronchodilators, whereas moderate exacerbations necessitated the use of systemic corticosteroids, antibiotics, or both. Severe exacerbations were marked by the need for an emergency department visit or hospital admission. This classification follows the criteria endorsed by the GOLD scientific committee. Non‐acute exacerbators (NAE) were defined as individuals who either did not experience any exacerbations or managed their symptoms solely with short‐acting bronchodilators.^21^

#### 2.2.4 IOS

The Master Screen IOS device (Viasys GmbH) measured respiratory resistance and reactance. Participants performed tidal breathing for 30–40 s through a mouthpiece. Following ERS guidelines, three tests were conducted, and the average values were recorded, including R5, R20, R5 − R20, X5, and Fres.

#### 2.2.5 FeNO, spirometry, and BDT measurements

FeNO (NIOX MINO, Aerocrine AB) was measured at a flow rate of 50 mL/s before spirometry. Spirometry and bronchodilator testing (BDT) were conducted by the same technologist using a Jaeger spirometer following ERS/ATS standards.

### 2.3 Statistical analysis

Data analysis was conducted using SPSS 26.0. The normality of data distribution was evaluated with the Kolmogorov–Smirnov test, and the homogeneity of variance was assessed using Levene's test. For data following a Gaussian distribution, results are presented as mean (SD) and non‐Gaussian data are expressed as median (IQR). Subsequently, independent sample *t*‐tests, Mann–Whitney tests, chi‐squared tests, or Fisher's tests were employed for inter‐group differential analysis of variables with continuous changes and categorical variable data. Spearman correlation analysis was conducted to assess the relationships between IOS parameters and pulmonary function indices, with the following correlation strength cutoff: 0 < |*r*| < 0.3 for weak correlations, 0.3 < |*r*| < 0.7 for moderate correlations, and |*r*| > 0.7 for strong correlations. Logistic regression and receiver operating characteristic (ROC) curve analysis were used to assess the diagnostic value of spirometry and IOS parameters. Differences in the areas under the ROC curves (AUCs) were compared using the DeLong test. A *p* values of <0.05 was considered statistically significant.

## 3 RESULTS

### 3.1 Part I

#### 3.1.1 Pathological manifestations of small airway

**FIGURE 1:**
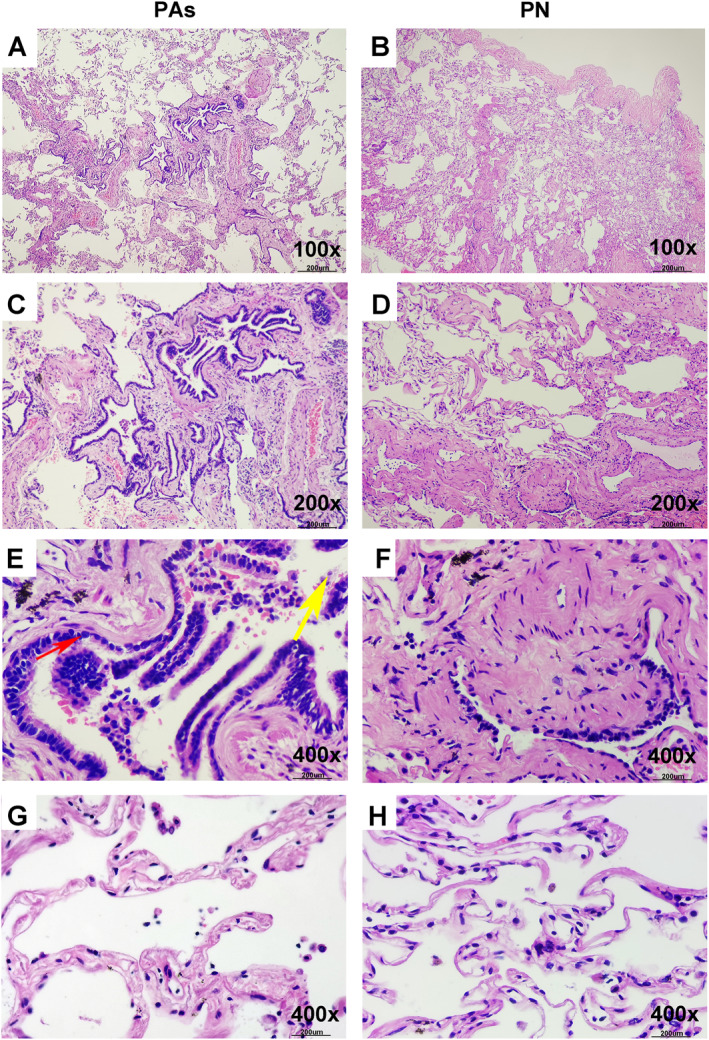
Representative photomicrographs of the lung are depicted. (A and B) Represent lower‐power views of (C–H), while (C–F) capture the small airways. (G and H) Display photomicrographs of alveoli with inflammatory cell infiltration in H&E‐stained sections. Macrophages and neutrophils are denoted by red and yellow arrows, respectively. Scale bars: 200 μm. H&E, hematoxylin and eosin; PAs, small airway pathological abnormalities; PN, small airway pathological normal.

**FIGURE 2:**
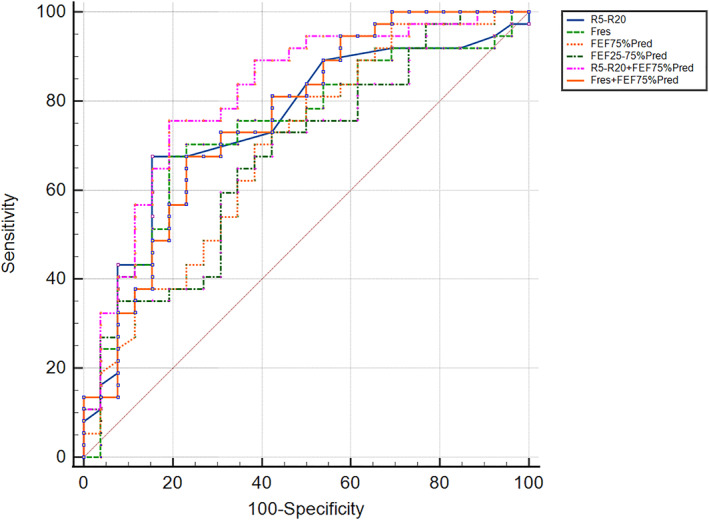
ROC curves of the joint models predicting PAs. *n* = 64; AUCR5 − R20 = 0.751 (95% CI, 0.626–0.851); AUCFres = 0.735 (95% CI, 0.609–0.838); AUCFEV_75_%pred = 0.693 (95% CI, 0.564–0.803); AUCFEV_25–75_%pred = 0.678 (95% CI, 0.548–0.790); AUCFres + FEV_75_%pred = 0.761 (95% CI, 0.637–0.859); AUC R5 − R20 + FEV_75_%pred = 0.816 (95% CI, 0.698–0.902). PAs, pathological abnormalities; ROC, receiver‐operating characteristic.

We collected 64 pathological tissue specimens from lung areas away from the lesions and conducted examinations. We analyzed the quantity of various inflammatory cell infiltration and assessed pulmonary interstitial spaces. The results indicate that early‐stage pathological abnormalities (PAs) in small airways are characterized by increased infiltration of macrophages, lymphocytes, neutrophils, and eosinophils as well as widening of the alveolar septa (Figure 1). By quantifying various infiltrating cells and alveolar septal distances, assigning scores, and calculating total scores, we divided the 64 patients into two groups: those with small airway PAs (*n* = 38) and those without (PN, *n* = 26). There were no differences in baseline demographic variables between the two groups. However, significant differences were found in spirometry (FEF_50_%pred, FEF_25–75_%pred) and IOS parameters (Fres, R5 − R20, R5). All demographic and clinical characteristics are detailed in Supporting Information S1: Table E1. Logistic regression analysis established a formula to predict PAs. As shown in Figure 2, R5 − R20 had a superior AUC compared to spirometry, with a cutoff value of 0.09. The highest diagnostic accuracy was achieved by combining R5 − R20 with FEF_75_%pred (AUC = 0.816, 95% CI: 0.698–0.902).

### 3.2 Part II

#### 3.2.1 Verification of R5 − R20 cutoff in monitoring COPD and ACO from healthy individuals

To validate the diagnostic accuracy of the R5 − R20 cut‐off values established in Part I in obstructive chronic airway diseases, a total of 37 healthy controls and 253 patients who met the criteria were included in this study, including COPD (*n* = 125), and ACO (*n* = 128). Statistical analysis showed no significant differences in sex, age, height, and body mass index (BMI) between patients and healthy controls. The baseline characteristics of these patients are shown in Table 1. Comparison of baseline pulmonary function between COPD and ACO showed no significant differences in large and small airway parameters. In the IOS parameters, although there were no notable differences in R5 − R20 and Fres between the two groups compared with healthy individuals, both cohorts demonstrated a median R5 − R20 surpassing 0.25 kPa/[L/s] and a median Fres exceeding 22 Hz. We utilized a cut‐off value of 0.09 kPa/[L/s] for the R5 − R20 to differentiate between a cohort of 37 normal individuals and 125 patients with COPD. The diagnostic evaluation of this cut‐off value showed a sensitivity of 85.6% and a specificity of 72.9%. The positive predictive value (PPV) was determined to be 91.4%, while the negative predictive value (NPV) was 60%. Furthermore, when the same cut‐off value of 0.09 for the R5 − R20 was used to differentiate between 37 normal individuals and 128 patients in ACO, the diagnostic performance showed a higher sensitivity of 93.75% with the same specificity of 72.9%. The PPV remained at 92.3%, while the NPV improved to 77.14%.

**TABLE 1:** Demographic data and clinical features of study participants in all subjects.

Characteristic variables	COPD (*N* = 125)	ACO (*N* = 128)	*p*‐value	Healthy control (*N* = 37)	*p*‐value
Age^a^	63 (9)	64 (15)	0.360	61 (24.5)	0.502
Sex: Male, *n* (%)^a^	94 (75.2)	89 (69.5)	0.330	23 (62.2)	0.27
Height, cm^b^	167.5 ± 8.1	167.6 ± 7.5	0.962	167.4 ± 8.2	0.977
BMI, kg/cm^2^ ^b^	23.4 ± 3.9	23.3 ± 3.2	0.894	23.7 ± 3.6	0.846
Former smoker, *n* (%)	73 (58.4)	71 (55.5)	0.677	11 (29.7)	0.007
Smoking pack‐years	33.25 ± 27.00	28.86 ± 23.35	0.410	6.53 ± 4.17	<0.001
Eosinophils, %^a^	2.05 (3.6)	3.5 (4.1)	**0.012**	1.8 (1.6)	<0.001
Eosinophils, ×10^9^/L^a^	0.15 (0.28)	0.22 (0.26)	**0.009**	0.13 (0.18)	<0.001
Neutrophils, %^a^	65.60 (15.18)	61.7 (13.8)	**0.039**	50.89 (13.69)	<0.001
Neutrophils, ×10^9^/L^a^	4.29 (2.35)	4.39 (2.59)	0.735	3.93 (2.06)	0.612
Lymphocytes, %^a^	22.35 (12.55)	25.6 (10.70)	**0.017**	34.5 (11.1)	<0.001
Lymphocytes, ×10^9^/L^a^	1.49 (0.96)	1.76 (0.88)	**0.010**	2.34 (1.44)	<0.001
Platelets, ×10^9^/L^a^	207.0 (70.25)	210 (100)	0.675	210 (77)	0.818
IgE, KU/L^a^	120 (233.8)	157.2 (620.83)	0.244	30 (33.6)	<0.001
FeNO, ppb^a^	18 (17)	28.5 (28.5)	**<0.001**	14 (5)	<0.001
R5 (kPa/[L/s])^a^	0.56 (0.30)	0.60 (0.33)	0.103	0.32 (0.04)	<0.001
R20 (kPa/[L/s])^a^	0.32 (0.12)	0.33 (0.13)	0.320	0.27 (0.03)	<0.001
X5 (kPa/[L/s])^a^	−0.23 (0.29)	−0.28 (0.30)	0.111	−0.06 (0.05)	<0.001
R5 − R20 (kPa/[L/s])^a^	0.25 (0.24)	0.28 (0.23)	**0.096**	0.043 (0.031)	<0.001
Fres (Hz)^a^	22.39 (7.44)	22.9 (10.25)	**0.092**	10.6 (0.390)	<0.001
FVC%pred^b^	73.2 ± 18.0	70.4 ± 14.9	0.270	96.0 ± 18.1	<0.001
FEV_1_%pred^b^	51.4 ± 17.3	48.8 ± 14.0	0.199	99.8 ± 16.1	<0.001
FEV_1_/FVC^b^	55.3 ± 10.1	55.1 ± 8.6	0.903	85.1 ± 6.2	<0.001
FEF_75_%pred^b^	21.7 ± 8.5	20.3 ± 8.0	0.168	91.1 ± 31.4	<0.001
FEF_50_%pred^b^	21.1 ± 10.8	19.7 ± 8.1	0.249	98.9 ± 19.0	<0.001
FEF_25–75_%pred^b^	21.4 ± 9.6	20.0 ± 7.8	0.208	94.9 ± 19.0	<0.001

*Note*: A trend toward difference was noted; however, it was not statistically significant.

Abbreviations: %pred, percent predicted; ACO, asthma‐COPD overlap; BMI, body mass index; COPD, chronic obstructive pulmonary disease; FEF_25–75_, FEF at 25%–75% of FVC; FEF_50_, FEF at 50% of FVC; FeNO, fraction of exhaled nitric oxide; FEV_1_, forced expiratory volume in 1 s; Fres, resonant frequency; FVC, forced vital capacity; pbb, parts per billion; R20, resistance at 20 Hz; R5, resistance at 5 Hz; R5 − R20, peripheral airway resistance difference between measurements at 5 and 20 Hz; X5, reactance at 5 Hz.

^a^ Median (interquartile range) values.

^b^ Mean ± SD values.

#### 3.2.2 Stronger correlation between IOS and spirometry in COPD compared to ACO across all severity levels

**FIGURE 3:**
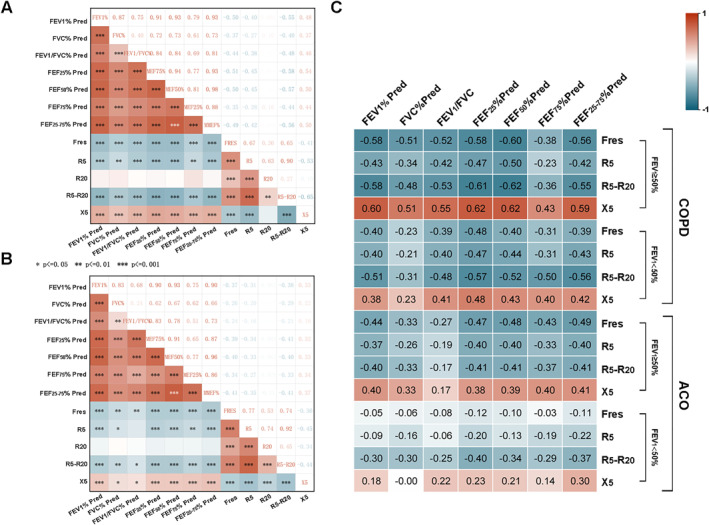
(A and B) Spearman correlation coefficient matrix and Spearman rank correlation tests. (C) Spearman correlation coefficient matrix and Spearman rank correlation tests in different FEV_1_%pred among the COPD and ACO groups. ACO, asthma‐COPD overlap; COPD, chronic obstructive pulmonary disease.

To further clarify the role of IOS in monitoring disease progression during follow‐up, 253 patients who met the criteria were included in this study, comprised by COPD (*n* = 125), and ACO (*n* = 128). The correlations between IOS and spirometry among the two groups are depicted in Figure 3. We can see that the baseline spirometry indicators are negatively correlated with Fres, R5, R5 − R20, and positively correlated with X5 in IOS among the two groups. In COPD, the correlations of FEV_1_, FEF_50_, and FEF_25–75_ with R5 − R20, X5 are the most substantial (ΙrΙ > 0.5) (Figure 3A). In the ACO group, the correlation coefficient was weaker (ΙrΙ = 0.4) (Figure 3B). We further subdivided the COPD and ACO groups at a threshold of FEV_1_%pred = 50%, creating two subgroups within each category. We then analyzed the correlations between various IOS parameters and spirometry indicators within these subgroups. In the COPD group, strong correlations were observed between IOS parameters and lung function measurements regardless of whether FEV_1_% was below or above 50%. Conversely, in the ACO group, stronger correlations were found in patients with Fres and FEV_1_%pred > 50% (ΙrΙ > 0.4) (Figure 3C).

#### 3.2.3 IOS combined with FEV_1_%pred, BMI, or FeNO can effectively prognosticate AE

Follow‐up evaluations were conducted for all patients, and the number of moderate to severe AE over the past year was recorded. A total of 140 patients were effectively followed up, comprising 66 patients in the COPD group and 74 patients in the ACO group, through both outpatient visits and telephone consultations. Within the COPD group, 19 patients experienced AE, while 47 did not. In the ACO group, 21 patients experienced AE, whereas 53 did not. Baseline analysis of relevant parameters was performed for both groups, with detailed data provided in Table 2.

**TABLE 2:** Demographic data and clinical features of 1 year follow‐up participants in the AE and NAE groups among COPD and ACO.

Characteristic	COPD		*p*‐value	ACO		*p*‐value
	AE group (*N* = 19)	NAE group (*N* = 47)		AE group (*N* = 21)	NAE group (*N* = 53)	
Male, *N* (%)	16 (84.2%)	34 (72.3%)	0.308	16 (76.2%)	40 (75.5%)	0.948
Age, years^a^	64 (11)	64 (10)	0.291	69 (8)	62 (15)	**0.014**
Height, cm^b^	167.6 ± 5.9	168.3 ± 9.0	0.818	168.9 ± 6.89	168.9 ± 6.90	0.991
BMI, kg/m^2^ ^a^	23.74 (9.66)	22.43 (4.88)	0.111	22.65 (6.56)	23.01 (4.84)	0.971
Smoking status, *N* (%)			0.332			0.631
Never smoker	5 (26.3%)	13 (27.7%)		6 (28.6%)	10 (18.9%)	
Ever smoker	3 (15.8%)	15 (31.9%)		7 (33.3%)	22 (41.5%)	
Current smoker	11 (57.9%)	19 (40.4%)		8 (38.1%)	21 (39.6%)	
FVC, %pred^b^	62.34 ± 11.98	78.96 ± 15.08	**<0.001**	60.71 ± 14.23	70.19 ± 14.69	**0.014**
FEV_1_, %pred^b^	40.83 ± 14.92	57.32 ± 16.41	**<0.001**	40.10 ± 9.21	49.72 ± 13.63	**0.004**
FEV_1_/FVC, %^b^	50.29 ± 11.26	57.19 ± 9.87	**0.016**	52.35 ± 8.72	56.24 ± 8.47	0.082
FEF_50%_, %pred^b^	15.64 ± 9.13	24.16 ± 11.34	**0.005**	14.57 ± 4.62	20.52 (7.88)	**0.018**
FEF_75%_, %pred^b^	16.69 ± 5.34	23.80 ± 8.69	**0.002**	16.40 ± 5.85	20.24 (6.45)	**0.020**
FEF_25%–75%_, %pred^b^	16.2 6 ± 7.93	24.23 ± 9.76	**0.003**	15.49 ± 4.68	20.61 (7.46)	0.005
R5, kPa/[L/s]^a^	0.61 (0.23)	0.50 (0.27)	**0.027**	0.73 (0.39)	0.58 (0.32)	**0.031**
R20, kPa/[L/s]^a^	0.32 (0.10)	0.32 (0.10)	0.955	0.32 (0.13)	0.32 (0.11)	0.513
Fres, Hz^a^	23.79 (5.94)	20.67 (9.07)	**0.027**	25.38 (12.76)	22.39 (7.82)	**0.042**
R5 − R20, kPa/[L/s]^a^	0.33 (0.19)	0.19 (0.18)	**0.001**	0.35 (0.24)	0.27 (0.22)	**0.010**
X5, kPa/[L/s]^a^	−0.29 (0.31)	−0.17 (0.20)	**0.008**	−0.34 (0.15)	−0.24 (0.28)	**0.009**
FeNO, ppb^a^	24 (16)	18 (18)	0.548	50 (74)	28 (27)	**0.042**
Inhaled medication			0.913			0.484
ICS/LABA/LAMA	13 (68.4%)	31 (66.0%)		11 (52.4%)	23 (43.4%)	
ICS/LABA	2 (10.5%)	4 (8.5%)		10 (47.6%)	30 (56.6%)	
LABA/LAMA	4 (21.1%)	12 (25.5%)				

*Note*: Statistical significance is shown by bold font.

Abbreviations: %pred, percent predicted; ACO, asthma‐COPD overlap; BMI, body mass index; COPD, chronic obstructive pulmonary disease; FEF_25–75_, FEF at 25%–75% of FVC; FEF_50_, FEF at 50% of FVC; FeNO, fraction of exhaled nitric oxide; FEV_1_, forced expiratory volume in 1 s; Fres, resonant frequency; FVC, forced vital capacity; ICS, inhaled corticosteroid; LABA, long‐acting beta‐agonist; LAMA, long‐acting muscarinic antagonist; pbb, parts per billion; R20, resistance at 20 Hz; R5, resistance at 5 Hz; R5 − R20, peripheral airway resistance difference between measurements at 5 and 20 Hz; X5, reactance at 5 Hz.

^a^ Median (interquartile range) values.

^b^ Mean ± SD values.

**FIGURE 4:**
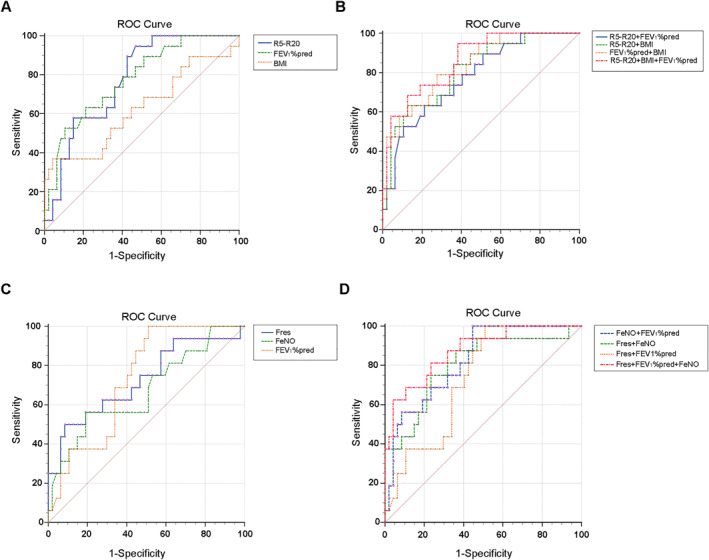
ROC curves of the models predicting positive AE. (A) ROC curves for R5 − R20, FEV_1_%pred, BMI to predict COPD AE, (B) is the pairwise combination of the indicators in (A) and the combination of the three indicators. (A) *n* = 66; AUCR5 − R20 = 0.775 (95% CI, 0.655–0.869); AUCFEV_1_%pred = 0.777 (95% CI, 0.658–0.870); AUCBMI = 0.626 (95% CI, 0.498–0.742); (B) AUCR5 − R20 + BMI = 0.811 (95% CI, 0.695–0.897); AUCR5 − R20 + FEV_1_%pred = 0.777 (95% CI, 0. 658–0.870); AUCFEV_1_%pred + BMI = 0.833 (95% CI, 0.721–0.914); AUCR5 − R20 + FEV_1_%pred + BMI = 0.860 (95% CI, 0.753–0.933); and (C) ROC curves for Fres, FEV_1_%pred, FeNO to predict ACO AE. (D) The pairwise combination of the indicators in (C) and the combination of the three indicators. (C) *n* = 74; AUCFres = 0.725 (95% CI, 0.598–0.830); AUCFEV_1_%pred = 0.731 (95% CI, 0.604–0.835); AUCFeNO = 0.672 (95% CI, 0.542–0.785) and (D) AUCFres + FeNO = 0.803 (95% CI, 0.684–0.893); AUCFres + FEV_1_%pred = 0.731 (95% CI, 0.604–0.835); AUCFEV_1_%pred + FeNO = 0.824 (95% CI, 0.708–0.909); AUCFres + FEV_1_%pred + FeNO = 0.874 (95% CI, 0.766–0.944). ACO, asthma‐COPD overlap; AE, acute exacerbation; BMI, body mass index; COPD, chronic obstructive pulmonary disease; ROC, receiver‐operating characteristic.

In patients with COPD, baseline spirometry values for both large and small airways demonstrated statistically significant differences between the AE group and the NAE group (*p* < 0.05). Except for R5, the remaining IOS parameters also showed differences between the two groups. Through logistic regression analysis, variables with a variance inflation factor (VIF) >10 were removed, and multivariable logistic regression analysis was performed on FEV_1_%pred, FEF_75_%pred, Fres, R5 − R20, X5, and BMI. The results indicated that R5 − R20, FEV_1_%pred, and BMI are significant factors influencing AE in COPD (Supporting Information S1: Table E2). The receiver operating characteristic (ROC) analysis revealed that a joint model of R5 − R20, FEV_1_%pred, and BMI exhibited the best predictive performance for patients at high risk of AE, with an AUC of 0.860 (0.753–0.933) (Figure 4A,B, Supporting Information S1: Table E4).

In patients with ACO, baseline lung function values, including FEV_1_%pred, FVC%pred, FEF_50_%pred, FEF_75_%pred, R5, R5 − R50, Fres, and X5, demonstrated statistically significant differences between the AE group and the NAE group (*p* < 0.05). Additionally, age and FeNO levels showed notable differences between the two groups. Logistic regression analysis was performed to remove variables with a VIF >10, and multivariable logistic regression analysis was ultimately conducted on FEV_1_%pred, FEF_75_%pred, Fres, R5 − R20, X5, FeNO, and age. The results indicated that Fres, FEV_1_%pred, and FeNO are significant factors influencing AE in ACO (Supporting Information S1: Table E3). The ROC analysis revealed that a joint model of Fres, FEV_1_%pred, and FeNO exhibited the best predictive performance for patients at high risk of AE, with an AUC of 0.874 (0.766–0.944) (Figure 4C,D, Supporting Information S1: Table E5).

## 4 DISCUSSION

Small airway dysfunction plays a pivotal role in the pathogenesis and progression of chronic airway diseases, particularly in COPD and ACO.^22^, ^23^ Although both conditions exhibit small airway involvement, their underlying pathophysiological mechanisms differ. IOS offers distinct advantages over traditional spirometry by detecting early SAD in an effort‐independent manner and being sensitive to peripheral airway changes often missed by conventional tests. Increasing evidence highlights its value in enhancing our understanding of airway diseases.^24^

With updated guidelines, the concept of pre‐disease, focusing on symptoms and structural abnormalities, has gained attention.^25^, ^26^ Cosio et al. found that nearly a quarter of those over 40 exhibited disease‐like symptoms and structural changes without spirometry‐detected obstruction.^25^ Therefore, in the first part of our study, we demonstrated that IOS effectively distinguishes patients with normal spirometry but underlying structural abnormalities, confirmed by microscopic analysis of pathological tissues from 64 patients. Logistic regression and ROC analysis revealed R5 − R20 (cutoff 0.09 kPa/[L/s]) outperformed FEF_75_%pred and other spirometry measures in detecting abnormalities. Combining R5 − R20 with FEF_75_%predicted further improved diagnostic accuracy, enhancing our ability to identify patients with these structural abnormalities earlier.

Current literature suggests using R5 − R20 values of 0.07 kPa/[L/s] as a diagnostic criterion for SAD, with FEF_25–75_%pred <65% as a Chiu et al.^27^ However, concerns about overdiagnosis with a fixed R5 − R20 cutoff of 0.075 kPa/[L/s] in healthy controls have been raised.^28^ Our previous research also found pathological small airway changes at an FEF_25–75_%pred cutoff of 80%,^16^ suggesting that the current threshold of R5 − R20 at 0.07 kPa/[L/s] may not accurately reflect small airway structural abnormalities, thereby highlighting the need for further evaluation of these diagnostic criteria. In addition, Chaiwong et al. studied the diagnostic role of IOS in COPD and chronic smoker populations, proposing a diagnostic cutoff of R5 − R20 ≥ 0.1 kPa/[L/s].^29^ The median pack‐year of smokers included in their study exceeded 30 pack‐years, suggesting that structural abnormalities may have already been present. Consequently, the R5 − R20 cutoff value established in our study, corroborated by pathological evidence, exhibits superior sensitivity compared to spirometry, offering more robust diagnostic criteria. Remarkably, this research is the inaugural confirmation of the early diagnostic benefits of the IOS parameter at the pathological level, coring its potential significance in clinical practice.

In the second part of our study, we enrolled 125 COPD patients, 128 ACO patients, and 37 healthy volunteers. Using an R5 − R20 cutoff of 0.09 kPa/[L/s], we found a PPV above 90% for identifying disease in the healthy group. Additionally, we reviewed the baseline data for R5 − R20 in COPD and discovered that some studies report a median of 0.19 kPa/[L/s], while others exceed this value.^30^, ^31^ Our data revealed a median R5 − R20 of 0.25 kPa/[L/s] in COPD and 0.28 kPa/[L/s] in ACO, with baseline R5 − R20 alone insufficient for a definitive diagnosis of airflow limitation. However, values above 0.2 kPa/[L/s] suggest possible airflow restriction, highlighting the need to refine bronchodilation tests. Next, we categorized COPD patients into mild to moderate and severe to very severe groups for the correlation analysis between spirometry and IOS parameters, finding correlation coefficients of 0.5 to 0.6 across all stages. This aligns with existing literature and emphasizes IOS's utility in long‐term follow‐up, particularly for elderly patients with severe disease.^32^, ^33^, ^34^ Similar correlations were seen in ACO, though slightly weaker. In COPD, R5 − R20 showed stronger correlations with spirometry than other IOS parameters, while Fres correlated more strongly in ACO. X5 correlations were weaker but more stable in patients with FEV_1_%pred below 50%. This may reflect the reduced small airway reversibility in COPD, while asthma's higher reversibility leads to lower IOS correlations. These differences highlight distinct SAD in each disease, offering insights for precision treatment.

Lastly, we conducted a retrospective analysis involving 140 patients who experienced AE within the preceding year. Our comparison of AE and NAE in COPD identified R5 − R20, FEV_1_%pred, and BMI as significant predictors through collinearity and multivariable logistic regression analyses. Although BMI did not show significant differences between groups, existing literature highlights its importance in acute COPD, prompting its inclusion in our analysis.^35^ ROC analyses of the three indicators, both independently and in combination, showed that the combined model achieved the highest AUC and significance. Notably, R5 − R20 and FEV_1_%pred produced comparable predictive AUCs, while combining BMI with R5 − R20 also resulted in a high AUC; however, FEV_1_%pred with BMI did not show significant differences. InACO, multivariable logistic regression identified Fres, FEV_1_%pred, and FeNO as predictors of AE, with the joint model yielding the highest AUC. Fres and FEV_1_%pred had similar independent predictive abilities, and combining Fres with FeNO resulted in a high AUC, whereas FEV_1_%pred with FeNO showed no significant difference. This reinforces Fres's predictive capacity for AE in ACO.^36^, ^37^ Our study further revealed that the differences in IOS parameters for predicting AE likely stem from the distinct pathophysiological features of COPD and ACO. In COPD, small airway obstruction is primarily due to inflammation, airway wall thickening, and parenchymal destruction, making R5 − R20, which monitors peripheral resistance, more meaningful. In contrast, ACO is characterized by airway remodeling, mucus hypersecretion, and complex inflammatory responses, which likely affect elastic resistance.^38^, ^39^ These findings provide deeper insights into the distinct aspects of the two diseases, contributing to a better understanding and improved management strategies.

Certainly, our study has several limitations. Although IOS can differentiate between healthy individuals and diseased patients, there is currently insufficient cutoff evidence to establish it as a definitive diagnostic tool for disease. As mentioned by Chaiwong et al., we plan to expand the sample size and incorporate post‐bronchodilator IOS to strengthen its role in diagnosing and managing chronic airway diseases and to validate its utility across different patient cohorts.

In conclusion, our study underscores the potential of IOS for the early diagnosis and management of COPD and ACO, showing advantages over traditional spirometry. The high diagnostic AUC of R5 − R20 (cutoff 0.09 kPa/[L/s]) supports IOS effectiveness in early detection. Additionally, R5 − R20 and Fres demonstrate predictive value for AE, reinforcing the routine use of IOS in clinical practice. Despite some limitations, our findings contribute to the growing evidence for integrating IOS into chronic airway disease management. With the increasing availability of portable IOS devices in primary care, our research provides valuable insights for improving patient care.

## AUTHOR CONTRIBUTIONS

**Yuning Huang**: Writing—original draft; investigation; methodology; formal analysis. **Xue Zhang**: Formal analysis; writing—original draft; methodology. **Jinwen Wang**: Software; formal analysis; investigation; visualization. **Wuping Bao**: Funding acquisition; formal analysis; validation. **Chengjian Lv**: Methodology; formal analysis; software. **Yingying Zhang**: Software; formal analysis; data curation. **Xue Tian**: Software; data curation; formal analysis. **Yan Zhou**: Funding acquisition; investigation; formal analysis; writing—review and editing. **Min Zhang**: Conceptualization; writing—review and editing; project administration; resources; supervision.

## CONFLICT OF INTEREST STATEMENT

The authors declare no conflicts of interest.

## Supporting information

Supporting Information S1

## Data Availability

The data that support the findings of this study are available on request from the corresponding author. The data are not publicly available due to privacy or ethical restrictions.
