# Supplementary material for: Role of impulse oscillometry in chronic obstructive pulmonary disease and asthma‐chronic obstructive pulmonary disease overlap
Source: Clin Transl Allergy. 2025 Apr 22;15(4):e70057. doi: 10.1002/clt2.70057 (PMC12012985; doi:10.1002/clt2.70057)
Supplement: Supplementary file 1 — Supporting Information S1 [file CLT2-15-e70057-s001.docx]

**Table E1.** Demographic data and clinical features of study participants in the PAs group and the PN group.

| **Characteristic variables** | **PAs (n=38)** | **PN (n=26)** | ***P*-value** |
| --- | --- | --- | --- |
| Age, years* | 58.13±7.491 | 54.08±10.14 | 0.075 |
| Male, n (%) † | 15.00(39.47) | 17.00(65.39) | 0.053 |
| Weight (kg) * | 64.37±9.948 | 66.35±10.60 | 0.457 |
| BMI * | 24.39±2.772 | 23.77±3.795 | 0.460 |
| Former smoker, n (%) | 9(23.68) | 6(23.07%) | 0.482 |
| Eosinophils (×10^9^/L) † | 0.120(0.09） | 0.100(0.120) | 0.551 |
| Eosinophils (%) † | 2.600(2.700) | 1.800(2.200) | 0.262 |
| Neutrophils (×10^9^/L) † | 2.670(1.340) | 2.930(1.240) | 0.494 |
| Neutrophils (%) † | 57.91±8.959 | 56.84±7.689 | 0.628 |
| Lymphocytes (×10^9^/L) * | 1.644±0.558 | 1.758±0.444 | 0.395 |
| Lymphocytes (%) * | 32.50±8.128 | 33.49±7.487 | 0.627 |
| Platelets (×10^9^/L) † | 194.0(51.00) | 172.0(92.00) | 0.216 |
| FeNO † | 31.00(18.00) | 19.00(23.00) | 0.122 |
| X5(kPa/[L/s]) † | -0.100(0.119) | -0.090(0.053) | 0.443 |
| Fres (Hz) † | 17.20(6.07) | 13.36(3.05) | **0.001** |
| R5(kPa/[L/s]) † | 0.433(0.232) | 0.313(0.183) | **0.028** |
| R20(kPa/[L/s]) † | 0.295(0.116) | 0.289(0.151) | 0.234 |
| R5-R20(kPa/[L/s]) † | 0.115(0.131) | 0.068(0.033) | **<0.001** |
| FEV_1_%pred * | 92.98±14.12 | 98.54±14.04 | 0.134 |
| FVC% * | 94.84±2.39 | 91.29±2.240 | 0.306 |
| FEV_1_/FVC * | 81.47±6.912 | 85.42±7.150 | **0.033** |
| FEF_50_%pred * | 77.20±24.61 | 91.30±23.03 | **0.026** |
| FEF_75_%pred | 55.73（42.69） | 90.57（78.16） | **0.006** |
| FEF_25-75_%pred * | 72.46±24.26 | 91.67±27.49 | **0.005** |

**Notes:** *Mean _ SD values.

†Median (interquartile range) values.

**Abbreviations**: BMI, body mass index; %pred, percent predicted; pbb, parts per billion; FeNO, fraction of exhaled nitric oxide; FEV_1_, forced expiratory volume in 1 second; FVC, forced vital capacity; IOS, impulse oscillometry; FEF_50_, FEF at 50% of FVC; FEF_25-75_,FEF at 25% to 75% of FVC; R5, resistance at 5 Hz; R20, resistance at 20 Hz; Fres, resonant frequency; X5, reactance at 5 Hz; R5-R20, peripheral airway resistance difference between measurements at 5 and 20Hz;

**Table E2.** Multivariable logistic regression of factors associated with COPD.

|  | **OR** | **95%CI** | ***P*-value** |
| --- | --- | --- | --- |
| R5-R20 | 1.654 | 1.003 to 2.725 | 0.048 |
| FEV_1_%pred | 0.931 | 0.880 to 0.984 | 0.011 |
| BMI | 1.334 | 1.094 to 1.626 | 0.004 |

**Table E3.** Multivariable logistic regression of factors associated in ACO.

|  | **OR** | **95%CI** | ***P*-value** |
| --- | --- | --- | --- |
| Fres | 1.135 | 1.020 to 1.263 | 0.020 |
| FEV_1_%pred | 0.915 | 0.848 to 0.987 | 0.021 |
| FeNO | 1.027 | 1.008 to 1.046 | 0.005 |

| **Predictive values** | **AUC** | **95% CL(AUC)** | **Sensitivity**  **%** | **Specificity**  **%** | **PPV**  **%** | **NPV**  **%** | ***P*-value** |
| --- | --- | --- | --- | --- | --- | --- | --- |
| R5-R20 | 0.775 | 0.655 to 0.869 | 94.74 | 53.19 | 45.00 | 96.2 | <0.001 |
| FEV_1_%Pred | 0.777 | 0.658 to 0.870 | 52.63 | 89.36 | 66.70 | 82.40 | <0.001 |
| BMI | 0.626 | 0.498 to 0.742 | 36.84 | 95.74 | 77.80 | 78.90 | 0.138 |
| R5-R20+BMI | 0.811 | 0.695 to 0.897 | 63.16 | 87.23 | 66.70 | 85.40 | 0.004 |
| R5-R20+ FEV_1_%pred | 0.777 | 0.658 to 0.870 | 52.63 | 89.36 | 66.70 | 82.40 | <0.001 |
| FEV_1_%Pred+BMI | 0.833 | 0.721 to 0.914 | 78.95 | 72.34 | 53.60 | 89.59 | <0.001 |
| **R5-R20+ FEV_1_%Pred+BMI** | **0.860** | **0.753 to 0.933** | **94.74** | **61.70** | **50.0** | **96.7** | **<0.001** |

**Table E4.** Predictive values in predicting AE of patients in COPD.

| **Predictive values** | **AUC** | **95% CL(AUC)** | **Sensitivity**  **%** | **Specificity**  **%** | **PPV**  **%** | **NPV**  **%** | ***P*-value** |
| --- | --- | --- | --- | --- | --- | --- | --- |
| Fres | 0.725 | 0.598 to 0.830 | 42.86 | 88.68 | 60.0 | 79.7 | 0.043 |
| FeNO | 0.672 | 0.542 to 0.785 | 56.25 | 80.85 | 50.0 | 84.4 | 0.040 |
| FEV_1_%Pred | 0.731 | 0.604 to 0.835 | 95.24 | 45.28 | 40.80 | 96.01 | <0.001 |
| Fres+FeNO | 0.803 | 0.684 to 0.893 | 75.00 | 76.00 | 52.20 | 90.00 | <0.001 |
| Fres+FEV_1_%pred | 0.731 | 0.604 to 0.835 | 95.24 | 45.28 | 40.80 | 92.6 | <0.001 |
| FEV_1_%Pred+FeNO | 0.824 | 0.708 to 0.909 | 100.0 | 55.32 | 43.2 | 100.0 | <0.001 |
| **Fres+FEV_1_%Pred+FeNO** | **0.874** | **0.766 to 0.944** | **62.50** | **95.74** | **83.3** | **88.2** | **<0.001** |

**Table E5.** Predictive values in predicting AE of ACO.
